# Commentary: Management of Intractable Pain in Patients With Implanted Spinal Cord Stimulation Devices During the COVID-19 Pandemic Using a Remote and Wireless Programming System

**DOI:** 10.3389/fnins.2021.696830

**Published:** 2021-08-05

**Authors:** Alessandro Dario, Giovanni Frigerio

**Affiliations:** ^1^Department of Neurosurgery, ASST Settelaghi, Insubria University, Varese, Italy; ^2^Pain Unit, Istituto Villa Aprica, Como, Italy

**Keywords:** spinal cord stimulation, telemedicine, trial, COVID-19, reimbursement

The article by Lu et al. (2020) regarding the use of a remote and wireless programming system with a spinal cord stimulation (SCS) device during the COVID-19 pandemic is very interesting since it focuses on the use of telemedicine with SCS in this particular period and, hence, opens up new scenarios in the field of neurostimulation.

We believe that the main points on the SCS management given by the pandemic are mainly two: first is the extensive use of telemedicine after having solved the regulatory problems and the education of the patients; and second is the possibility to perform with the one-shot implant.

In particular, before the pandemic, the use of telemedicine was quite limited in neurosurgical practice (Basil et al., 2021).

On the other hand, two neurostimulator implants are required for a successful application: the trial implant and the definitive implant. This temporal procedure may be limited by the current demands that the health system faces to combat the pandemic.

In two experienced SCS centers in Lombardy, due to the restrictions of social contact during the epidemic, the follow-up in the trial period between the SCS programmer doctor and the patient was largely by telephone or video system. However, the novelty of the new system, the inability to use it by elderly people, and the difficulty of communication caused relationship problems between the doctor and the patient. Hence, out of 31 patients with SCS trials, 5 patients could not get an indication from the trial because it was not actually performed in their case (Dario et al., 2021).

## Telehealth and Scs

The use of telemedicine in SCS implantation-indication as reported is rather low (Basil et al., 2021), but it is during the trial or immediately after the definitive implantation that telemedicine can almost completely replace face to face visits (Lu et al., 2020). This method can be an innovation in the management of SCS for chronic pain and it must be used to offer the best experience for patients with chronic pain (Marinangeli et al., 2020).

Worldwide, the use of telemedicine systems is still lagging (Golinelli et al., 2020). In Italy, the “Telemedicine National Guidelines” recognizes the substantial equivalence between the services provided in telemedicine and traditional service. The integration of traditional services and telemedicine should be framed organically within diagnostic-therapeutic-assistance paths (Ferorelli et al., 2020). Moreover, the report provided by the National Health Institute in April 2020 entitled “Interim indications for telemedicine assistance services during the COVID-19 health emergency” proposed the implementation of telemedicine in subjects suffering from chronic disease or in frail conditions: the chronic pain can be considered a chronic disease that necessitates continuous health monitoring since spontaneous recovery is rare (Eccleston et al., 2020).

Unfortunately, the speed of pandemic expansion in Italy did not allow for the adequate development of telemedicine services in relation to the SCS and, above all, the adequate integration of the regulatory framework throughout the national territory. On the other hand, Italian telemedicine services concerning cardiology, pulmonology, and neurology are already being implemented before the pandemic (Cilia et al., 2020; De Simone et al., 2020). As a first step in telemedicine, a telephone call with answers to simple and clear questions about the course of pain in patients with SCS may be appropriate (Emerick et al., 2020).

## Trial or No Trial

Recently, the need for a trial prior to implantation of the definitive neurostimulator has been questioned (Chadwick et al., 2020) since the outcomes on the efficacy with respect to the distance of SCS are similar both in patients using the trial and in those who are not using it. Moreover, considerable savings could be demonstrated by the use of an implantation only without a screening trial (Eldabe et al., 2020). Furthermore, the adoption of neurostimulators capable of using multiple waveforms has increased the positive results (Haider et al., 2018; Fishman et al., 2020) and the use of devices with an external IPG has eliminated the need for an implantable battery (Ahmadi et al., 2021).

## Discussion

At the moment, telemedicine is a necessity during the pandemic. However, it could also be an improvement for the future management of SCS. Our experience was not completely positive, but this was due to the fact that, in a short time, we had to adapt to the emergency of the pandemic with a relatively low number of implants. Moreover, telemedicine provides for an experience both by the doctor and the patient or their family members. SCS telemedicine requires the patient or family members to know how to use web links. This may be the most critical point of the whole procedure; failure to connect via the web can increase the sense of abandonment of the patient as experienced by some of our patients, who then rejected the definitive implant. In many countries, older patients could need the presence of a family member or a caregiver to use the internet. The satisfaction with teleconsultations is reported to be high and 45% of doctors plan to continue telemedicine after the epidemic has waned (Tandon et al., 2021).

However, the problem on how to obtain the appropriate reimbursement for visits that are made online remains. Moreover, there is a need for legislation and medicolegal value focused on SCS telemedicine (Blue et al., 2020).

SCS telemedicine is an opportunity that can be enhanced by the use of devices that offer different neurostimulation waves or that can be implanted with a single intervention. In fact, new waveforms and multiple programs help because they offer more programs to test and, therefore, get the most suitable positive result for the patient (Haider et al., 2018).

We congratulate Doctor Lu and colleagues for demonstrating the advances in SCS telemedicine, and we hope that this method will be applicable to all spinal neurostimulation devices.

Moreover, the possibility of implanting the whole system in a hospital intervention in a single day is another encouraging factor in the health system, which is currently overwhelmed by the COVID-19 pandemic. However, the one-shot implant should be reserved for those patients who meet the appropriateness criteria for SCS (Deer et al., 2014).

In conclusion, the impact of COVID-19 on SCS procedures may have been negative but the pandemic has offered opportunities to change the clinical and economic management of the implants themselves and has alternative options for progress in the use of this technique. Two seem to be the key points of this progress: the use of telemedicine and the one-shot SCS implant.

## Author Contributions

AD and GF designed and conceptualized this work and participated in drafting the manuscript. Both authors contributed to the article and approved the submitted version.

## Conflict of Interest

The authors declare that the research was conducted in the absence of any commercial or financial relationships that could be construed as a potential conflict of interest.

## Publisher's Note

All claims expressed in this article are solely those of the authors and do not necessarily represent those of their affiliated organizations, or those of the publisher, the editors and the reviewers. Any product that may be evaluated in this article, or claim that may be made by its manufacturer, is not guaranteed or endorsed by the publisher.
